# Linker-Determined Folding and Hydrophobic Interactions Explain a Major Difference in PROTAC Cell Permeability

**DOI:** 10.1021/acsmedchemlett.5c00068

**Published:** 2025-03-17

**Authors:** Vasanthanathan Poongavanam, Stefan Peintner, Yordanos Abeje, Florian Kölling, Daniel Meibom, Mate Erdelyi, Jan Kihlberg

**Affiliations:** † Department of Chemistry - BMC, 99028 Uppsala University, Box 576, 75 123 Uppsala, Sweden; ‡ Drug Discovery Sciences, Bayer AG, 42113 Wuppertal, Germany

**Keywords:** PROTAC, cell permeability, MD simulations, NMR spectroscopy, hydrophobic collapse

## Abstract

The ability to adopt folded conformations that have a low solvent-accessible 3D polar surface area has been found to be important for PROTACs to display a high passive cell permeability. We have studied two VHL PROTACs that differ only by the replacement of two methylene groups in the linker by oxygen atoms but that displayed vast differences in their cell permeability. MD simulations and NMR spectroscopy revealed an unexpected, environment-dependent conformational behavior for the low-permeability PROTAC that has an alkyl linker. Hydrophobic interactions enforced extended and polar conformations for this PROTAC in nonpolar media, explaining its low cell permeability. In water, hydrophobic collapse around the linker led to folded and less polar conformations. In contrast, the highly permeable PROTAC having a PEG linker adopted conformations of similar shapes and polarities in polar and nonpolar environments.

## Graphical abstract

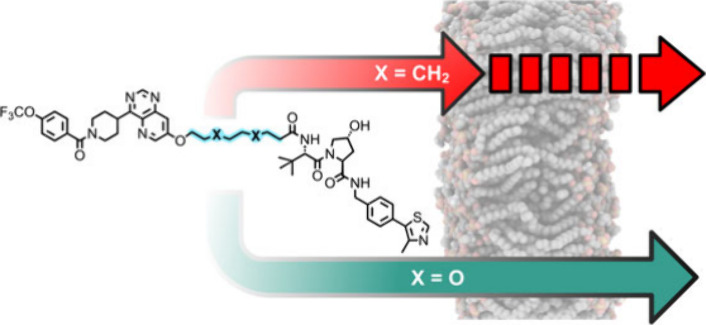

Solubility, cell permeability, and first-pass metabolism in the liver are the three most important determinants of oral bioavailability. As a consequence of their heterobifunctional structure, proteolysis-targeting chimeras (PROTACs)^1,2^ reside in the beyond rule of 5 (bRo5) chemical space,^3–5^ where molecular size, polarity, and/or lipophilicity often makes it challenging to achieve satisfactory bioavailability.^6,7^ PROTACs based on a von Hippel–Lindau (VHL) E3 ligase ligand are located farther into the bRo5 space than PROTACs based on ligands for cereblon (CRBN) and thus carry a larger risk of not being orally bioavailable.^8^ Moreover, since the structure and properties of the ligand of the target protein of interest (POI) are determined by the binding site on the POI, the choice and optimization of the linker of VHL PROTACs intended for oral administration requires great care.^9,10^

2D descriptors, such as those of Lipinski’s^11^ and Veber’s^12^ rules, provide a first indication of the likelihood that a PROTAC will be orally bioavailable. However, 2D descriptors are not optimal for the identification of oral PROTACs since they place CRBN and VHL PROTACs in different parts of chemical space, even when the members of the two classes display similar bioavailabilities.^8^ Instead, recent studies based on NMR spectroscopy, high-throughput chromatographic methods, and/or in silico predictions have found that conformation-dependent 3D descriptors of size and polarity correlate better to cell permeability for PROTACs than traditional 2D descriptors.^8^ Herein, we have used unrestrained MD simulations followed by NMR-based conformational studies to provide a detailed mechanistic understanding of how a minor difference in the structure of the linker of two otherwise identical VHL PROTACs translates into major differences in conformational behavior and a large difference in cell permeability.

PROTACs **1** and **2** have flexible aliphatic and ethylene glycol-based linkers connecting the extracellular signal-regulated kinase 5 (ERK5)^13^ and VHL ligands and molecular weights just under 1000 Da (Figure 1). We recently reported that this seemingly minor structural difference between the linkers is associated with a close to 3 orders of magnitude higher permeability for **2** than for **1** in the parallel artificial membrane permeability assay (PAMPA).^14^ As judged by the ratio of the potencies for binding to VHL in a cell-based assay and a biochemical assay, **2** is 22-fold more cell-permeable than **1**. Attempting to understand the origin of the permeability differences between **1** and **2**, we first turned our attention to their physicochemical properties.

**Figure 1:**
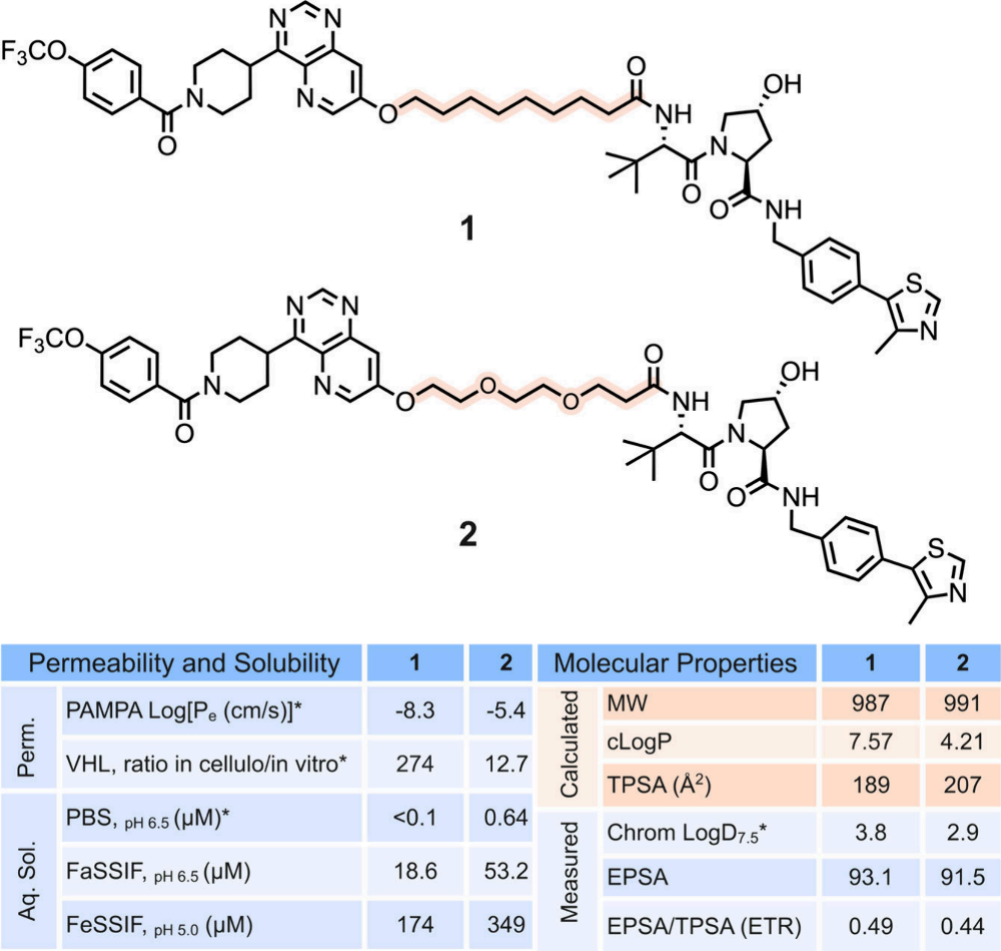
Structures, membrane permeabilities, aqueous solubilities, and calculated and measured properties for PROTACs **1** and **2**. The difference in the linkers between **1** and **2** is indicated by orange shading. *Data reported recently.^14^

The solubilities of **1** and **2** are very low in PBS but substantially higher in the biologically more relevant fasted- and fed-state simulated intestinal fluids (FaSSIF and FeSSIF, respectively) (Figure 1). While the solubilities of **1** and **2** are similar in the three assays, it is noteworthy that the more permeable **2** consistently has a solubility somewhat higher than that of **1**. We conclude that the differences in permeability between **1** and **2** do not originate from a too-low solubility or from differences in solubility.

The calculated lipophilicity (cLogP) is more than 3 orders of magnitude higher for the less permeable **1** than for **2**, but their chromatographic LogD values differ by less than 1 order of magnitude and fall in the higher end of the “drug-like” range (Figure 1). Having a higher chromatographic LogD, **1** would be expected to be the more permeable of the two, contrary to the experimental permeability data. Lipophilicity thus does not appear to be the immediate reason for the difference in permeability between the two PROTACs. As expected from the structural difference in the linkers of **1** and **2**, the topological polar surface area (TPSA) is somewhat larger for **2**, suggesting **2** to be the more polar of the pair. The chromatographically determined measure of polarity, the experimental polar surface area (EPSA),^15^ differs in the opposite direction between the two PROTACs, with **2** being slightly less polar than **1**. Consequently, the EPSA to TPSA ratio^16^ (ETR) is also somewhat lower for **2** than for **1**. The EPSA and ETR values of **1** and **2** indicate that both PROTACs fold and hide a substantial degree of polarity in a nonpolar environment, potentially by behaving as molecular chameleons.^17^ However, the EPSA and ETR values of **1** and **2** differ only marginally and do not provide any rationalization for the large differences in the permeability between the two PROTACs.

Having concluded that the differences in permeability between **1** and **2** do not correlate to their physicochemical properties, we performed unrestrained MD simulations in explicit chloroform and water. Chloroform (ε = 4.8) was used because it has a dielectric constant close to that of the interior of a cell membrane (ε = 3.0)^18^ and to allow comparison to our recent NMR studies.^14^ The simulations converged within 5–10 ns, and the variation between the replicates was small to moderate (Figure S1). Boltzmann population analysis provided an understanding of the low-energy conformational landscape for each PROTAC in the two environments.

The permeability of a compound across a cell membrane depends on its size and polarity.^19^ For compounds within the realm of Lipinskís rule of 5, size is described by molecular weight (MW) and polarity by hydrogen-bond acceptor (HBA) and hydrogen-bond donor (HBD) counts.^11^ For larger and more complex compounds in the bRo5 space, it has been found that size is better approximated by the radius of gyration (*R*_gyr_),^19,20^ while the solvent-accessible 3D polar surface area (SA 3D PSA) has become an established descriptor of polarity.^19,21^ Analysis of the MD simulations using these two descriptors suggested that **1** and **2** populated similar property spaces in water (Figure 2, left panels). The most sampled regions for both **1** and **2** had a low *R*_gyr_ (∼5–7 Å), characteristic of ensembles of folded and semifolded conformations, while the SA 3D PSA was centered at 220 Å^2^. The low-energy conformations and the minimum-energy conformations (MECs) of both PROTACs were also found in similar conformational spaces located adjacent to the most sampled regions, i.e., with the MECs at *R*_gyr_ just over 5 Å and SA 3D PSA close to 190 Å^2^ (Figure 2).

**Figure 2:**
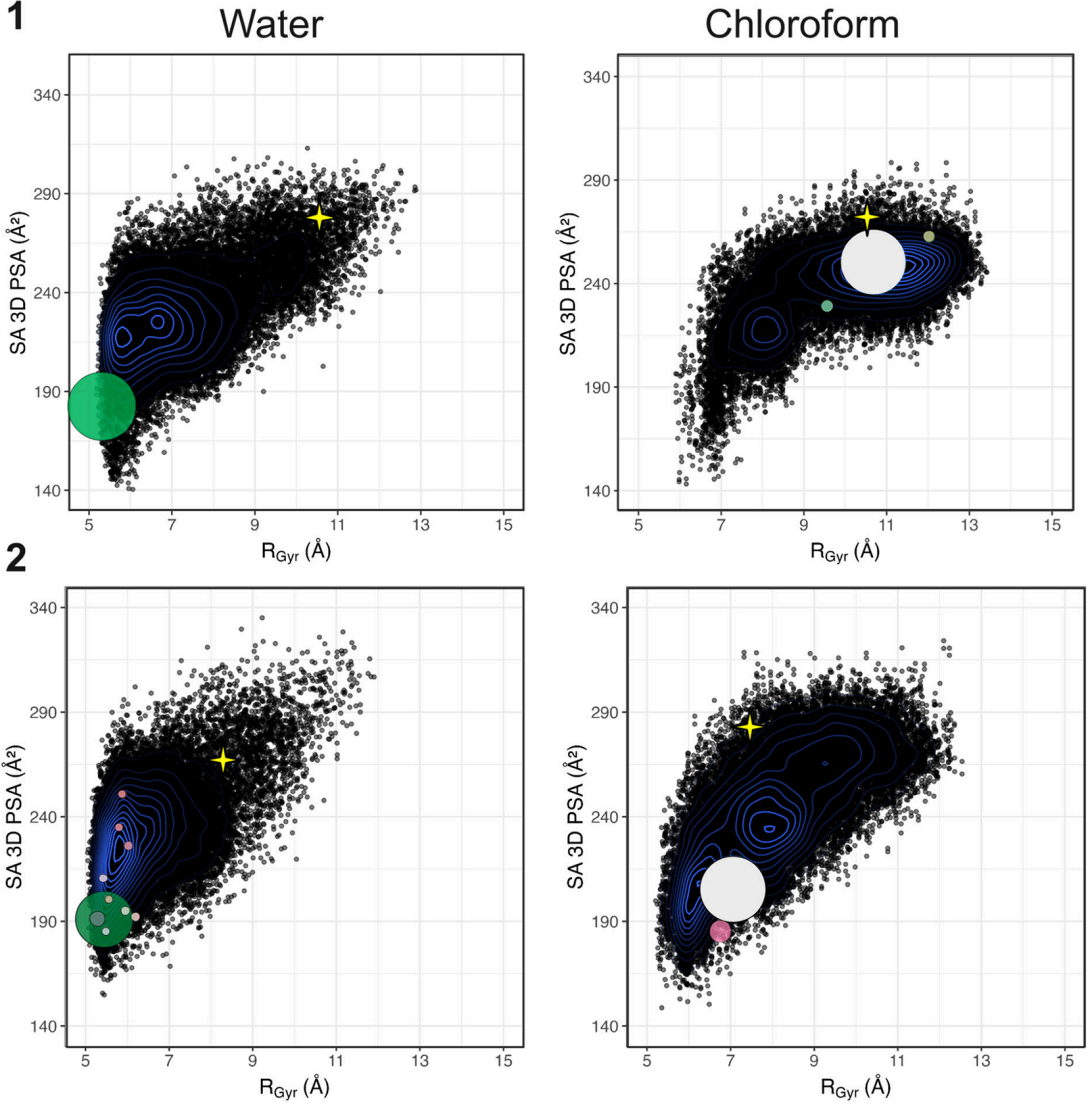
Solvent-accessible 3D polar surface area (SA 3D PSA) versus the radius of gyration (*R*_gyr_) of the conformations from the MD simulations of PROTACs **1** and **2** in explicit water and chloroform. Densely sampled property space is indicated by the landscape contours. Circles show the location of low-energy conformations having a population of >1% after clustering and Boltzmann population analysis. The area of each circle is proportional to the population of the corresponding conformation, and thus, the largest circle identifies the minimum-energy conformation (MEC). The yellow star indicates the starting conformation for each MD simulation.

When transitioning from water into chloroform, the most sampled property space of **1** shifted from folded and semifolded conformations in water to extended conformations in chloroform (*R*_gyr_ ∼ 11 Å; Figure 2, top right). Simultaneously, and perhaps counterintuitively, the SA 3D PSA of the frequently sampled conformations increased from ∼ 220 Å^2^ in water to just over 240 Å^2^ in chloroform. The MEC of **1** in chloroform is also located in this highly sampled space of extended and polar conformations, far from the folded and less polar MEC in water. For **2**, the transition from water to chloroform resulted in a shift of the most sampled space from being predominantly folded to an ensemble dominated by both folded and semifolded conformations (*R*_gyr_ ∼ 5.5 → 6 and 8 Å; Figure 2, bottom). The folded conformations in chloroform are slightly less polar than those in water (SA 3D PSA ∼ 210 versus ∼ 220 Å^2^, respectively), while the semifolded ensemble is more polar (SA 3D PSA ∼ 240 Å^2^). The MEC of **2** in chloroform is found in a similar but somewhat more polar and elongated property space than the MEC in water. In summary, both the frequently sampled property spaces and the location of the MECs reveal a conformational transition whereby **1** unfolds and adopts a more polar ensemble as it transitions from water to chloroform or from water to the interior of a cell membrane. The large *R*_gyr_ and high SA 3D PSA of the chloroform ensemble of **1** rationalize its very low membrane permeability. For PROTAC **2**, the property spaces populated in water and chloroform, and the MECs in the two solvents, are much more similar than for **1**. The small *R*_gyr_ and low SA 3D PSA of the ensemble of **2** in chloroform agree well with its high cell permeability.

The distribution of a compound between environments that differ in polarity and the conformational ensembles populated in each environment are also influenced by hydrophobic interactions involving the lipophilic moieties of the compound.^19^ To gain a deeper understanding of the conformational behavior of **1** and **2**, we also calculated the solvent-accessible 3D nonpolar surface area (SA 3D NPSA) of their low-energy conformations (Figure 3 and Table S1). The SA 3D NPSAs of the MECs of **1** and **2** illustrate why **1** adopts a more extended and more polar conformation in chloroform than in water and also provide additional insight into the large difference in permeability displayed by the two PROTACs. In water, the MEC of **1** has undergone a hydrophobic collapse^22^ so that the linker is located in a groove formed by parts of the ERK5 and VHL ligands (Figure 3A, left). Even though this MEC has a lower SA 3D PSA and contains an additional intramolecular hydrogen bond (IMHB) compared to the MEC in chloroform, the driving force to minimize exposure of the aliphatic linker in water, i.e., to minimize the SA 3D NPSA, appears to have a strong influence on the conformational preference of **1**. Solvation of the aliphatic linker in chloroform results in a MEC that has substantially increased SA 3D NPSA but also exposes the polar moieties in the ERK5 and VHL ligands to a larger extent than in water. PROTAC **2** behaves differently, with the MEC in water being folded while the one in chloroform is semifolded (Figure 3B). However, the two MECs have similar SA 3D PSAs and SA 3D NPSAs, with the PEG linker being solvent-exposed. The conformations of the MECs thus point to the hydrophobic collapse of **1** around the aliphatic linker in water and exposure of its polar groups in chloroform driven by solvation of its aliphatic linker as the reasons for its low permeability. In contrast, the MECs of **2** reside in similar property space in water and chloroform, rationalizing its high cell permeability. The conformational behavior of PROTAC **2** shows similarities to compounds that populate congruent conformations, i.e., conformations which are identical in water and in the cell membrane. Adoption of congruent conformations has been reported to be essential for macrocyclic peptides to achieve high cell permeability.^23^

**Figure 3:**
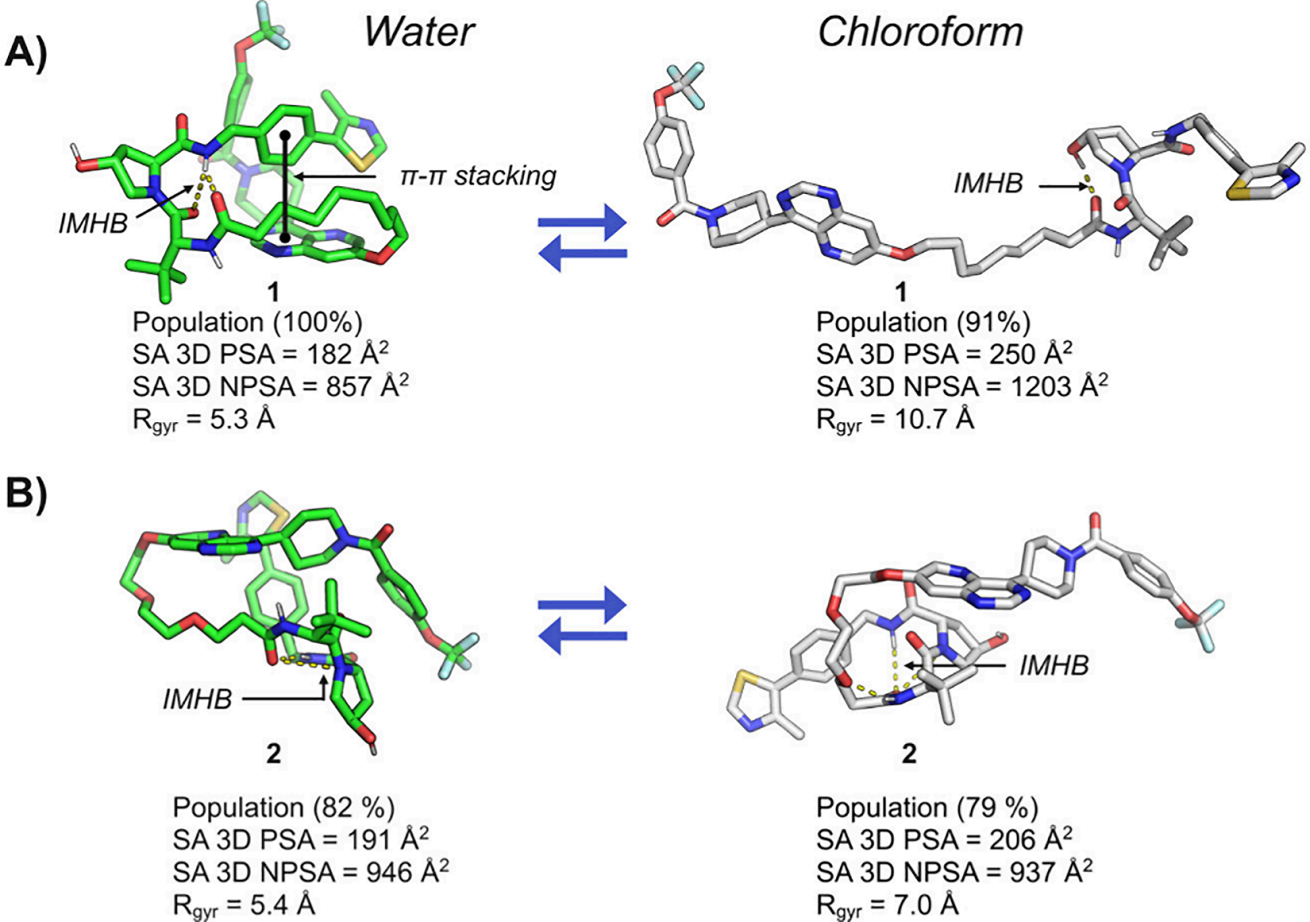
(A, B) Minimum-energy conformations (MECs) from the MD simulations in explicit water and chloroform for **1** and **2**, respectively. The solvent-accessible 3D polar surface area (SA 3D PSA), solvent-accessible 3D nonpolar surface area (SA 3D NPSA), and radius of gyration (*R*_gyr_) are given below each conformation.

Intrigued by the somewhat counterintuitive conformations predicted for **1** in water and chloroform and also by the similarities between the conformations of **2** in the two environments, we extended our recent NMR studies of **1** and **2** in chloroform^14^ to an aqueous environment. Due to the low solubilities of the two PROTACs in PBS (Figure 1), a DMSO/water mixture was used instead of water. ^1^H NMR spectra were recorded at low temperature to obtain optimal chemical shift dispersion, and then NOEs were identified, which provide information about the shape of the conformations in the ensembles (Figure 4). To provide detailed insight into the ensemble populated by **1** in DMSO/water, its NOEs were further deconvoluted into atom-level-resolved conformations using the NMR analysis of molecular flexibility in solution (NAMFIS) algorithm.^24^

**Figure 4:**
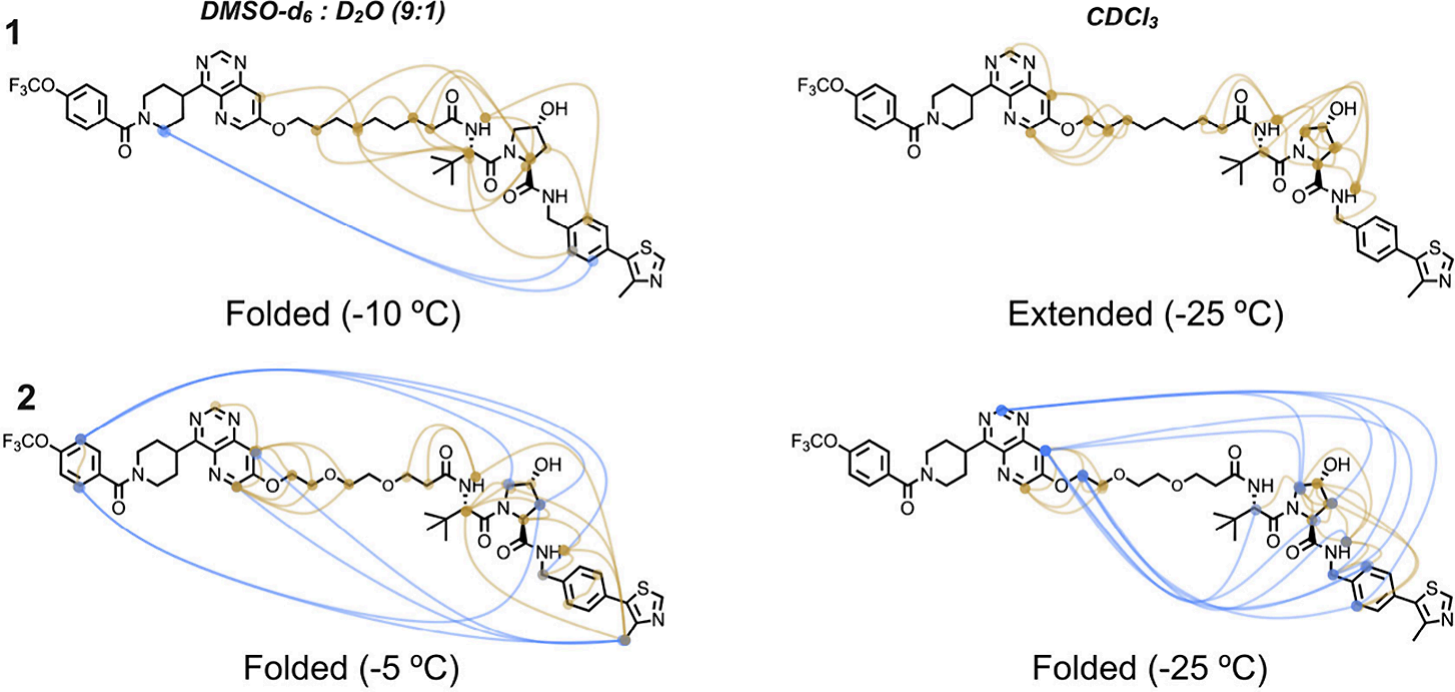
Overview of the experimentally determined nuclear Overhauser effects (NOEs) that were used in the NAMFIS analysis of PROTACs **1** and **2**. The shape of the conformations in each ensemble that can be inferred from the NOEs is stated below the structures of the PROTACs, with the temperature used to record the NMR spectra given in parentheses. Blue lines indicate long-range NOEs between protons in the ERK5 and VHL ligands or between protons in one of the ligands and protons at the other end of the linker. All other medium- and short-range NOEs are indicated by brown lines.

In DMSO/water PROTAC **1** displays two long-range NOEs between the two ligands, as well as some medium-range NOEs between protons in the part of the linker adjacent to the ERK5 ligand and in the *tert*-butylglycine moiety of the VHL ligand. This NOE pattern suggests that the conformational ensemble of **1** mainly consists of folded and semifolded conformations in the polar environment. The lack of long- and medium-range NOEs in chloroform is indicative of extended conformations in a nonpolar environment. The large number of long-range NOEs found for PROTAC **2** in DMSO/water and in chloroform reveals that **2** mainly adopts folded and semifolded conformations both in polar and nonpolar environments. The use of DMSO/water as solvent in the NMR studies constitutes a limitation when compared to the MD simulations, which were performed in water. However, the agreement in the overall shapes of the predicted and experimental conformational ensembles is strong. Thus, both MD simulations and NMR spectroscopy find that **1** adopts folded conformations in polar environments (water and DMSO/water, respectively) but extended ones in chloroform, whereas **2** has a high degree of folding in both environments (Figures 2–4).

NAMFIS analysis of **1** in DMSO/water provided an ensemble in which five conformations, representing 54% of the ensemble, are folded (Figures 5A and S10). Similarly, the MEC from the MD simulations and the conformations in the most frequently sampled space in water are folded (Figures 2 and 3). Interestingly, the most populated conformation from the NAMFIS analysis (conf 4) has a cylindrical fold, with the aliphatic linker located in a nonpolar groove formed by parts of the ERK5 and VHL ligands (Figure 5B), i.e., a conformation with a hydrophobic collapse similar to that in the MEC from the MD simulations (Figure 3A). One of the minor conformations (conf 3, 6%, Figure S10) shows a hydrophobic collapse around the linker similar to that of the most populated conformation. Minor conformation 10 (3%) displays a different form of hydrophobic collapse (Figure 5B) in which the phenyl rings of the ERK5 and VHL ligands form a π–π interaction, with the linker running across the top of the π–π complex. The DMSO/water ensemble of **1** also consists of four semifolded conformations (39%, exemplified by 9, Figure 5B) and one extended conformation (2, 7%). With some exceptions both the SA 3D PSA and SA 3D NPSA increase in going from folded to semifolded and extended conformations (Table S7).

**Figure 5:**
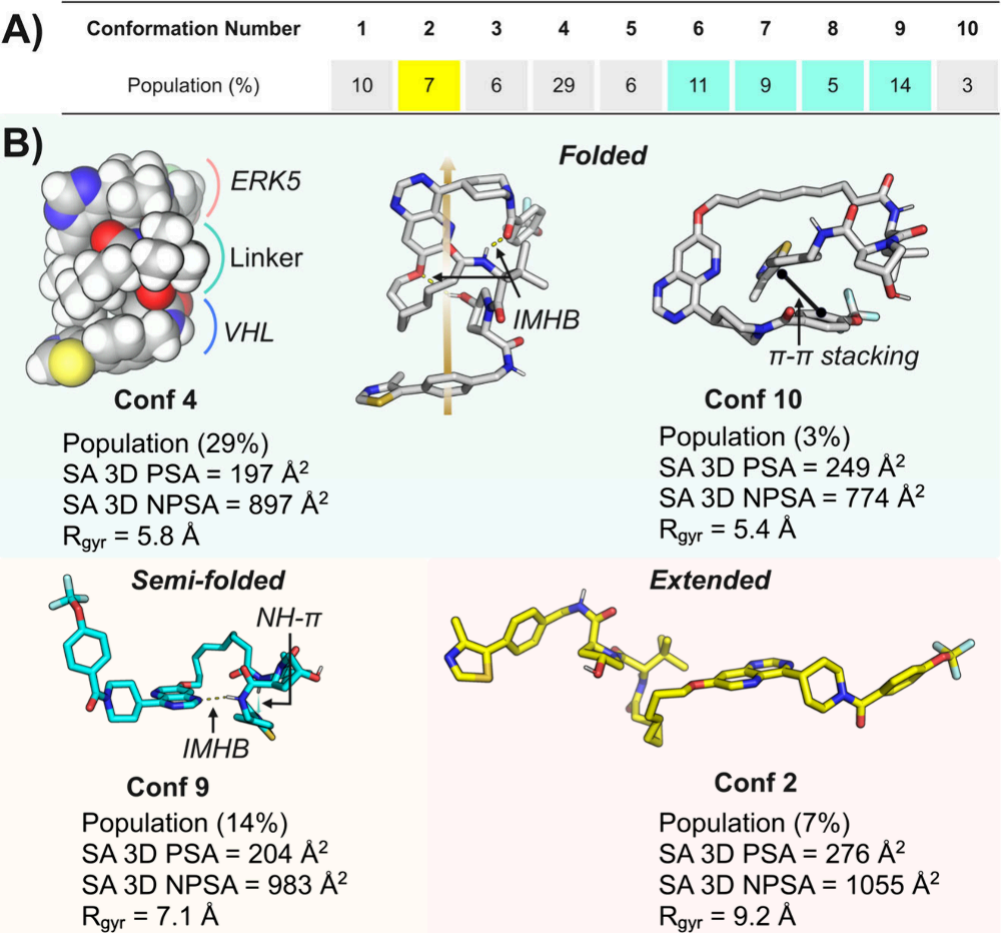
(A) Populations of the conformations in the ensemble determined by NAMFIS analysis for **1** in 9:1 DMSO-*d*_6_/D_2_O at −10 °C. The color coding indicates the folding of each conformation: gray, folded; cyan, semifolded; yellow, extended. (B) Surface and stick representations of representative conformations of **1**, i.e., the most populated folded (conf. 4), semifolded (9), and extended (2) conformations, and a minor folded conformation (10). The yellow arrow indicates the axis through the cylindrical conformation 4. The solvent-accessible 3D polar surface area (SA 3D PSA), solvent-accessible 3D nonpolar surface area (SA 3D NPSA), and radius of gyration (*R*_gyr_) are given below each conformation. The SA 3D PSA was calculated with VEGA ZZ (version 3.2.3), as for the MD simulations (cf. Figures 2 and 3), which required a high-throughput method, instead of using our previously reported protocol^25^ based on PyMOL. The two methods are strongly correlated (*R* ^2^ = 0.94), but values from VEGA are higher than those from PyMOL (Figure S11). Oxygen atoms are red and nitrogen atoms blue, while the sulfur atom is yellow.

The solubility of conformationally flexible compounds, such as PROTACs, is influenced by driving forces that expose polar moieties to water and hide nonpolar moieties by hydrophobic collapse in an aqueous environment. Conversely, the shielding of polar moieties by the formation of intramolecular interactions and the exposure of nonpolar moieties in a membrane contribute to high passive cell permeability. Our studies of VHL PROTACs **1** and **2** illustrate how this delicate balance of inter- and intramolecular interactions is influenced in a dramatic way by the replacement of two methylene groups in the aliphatic linker of **1** by oxygen atoms to give **2** and how the resulting conformational preferences of the two PROTACs explain the large difference in permeability between them. Neither 2D descriptors, e.g., those of Lipinski’s and Veber’s rules, nor experimentally determined physicochemical properties were able to provide this insight.

Previously, molecular chameleonicity,^17^ i.e., the ability of a compound to dynamically expose or shield polar functionalities in response to the properties of the environment, has been highlighted as a mechanistic explanation for how PROTACs can achieve high cell membrane permeability.^8,26–28^ Herein, we identified the ability to adopt conformations that have similar 3D descriptors of shape and polarity as another mechanism by which VHL PROTACs such as **2** can display high membrane permeability. In addition, we could trace the low permeability of **1** to the rebalancing of hydrophobic inter- and intramolecular interactions of the aliphatic linker in environments that differ in polarity. In an aqueous environment, hydrophobic collapse around the linker results in **1** adopting folded conformations, while solvation of the linker in nonpolar environments leads to an ensemble of extended conformations in a membranelike environment. Due to the solvation of the linker, these extended conformations also expose the polar groups of **1**; both shape and high polarity contribute to the low membrane permeability of **1**. The importance of the folding mechanisms reported herein merits further exploration beyond those of PROTACs **1** and **2**. We speculate that folding may be more important for orally bioavailable VHL PROTACs, among which flexible linkers currently dominate, than for CRBN PROTACs, which have more rigid linkers.^8^

This and previous investigations of CRBN PROTACs reveal that MD simulations in explicit solvents provide conformational ensembles that overlap with those determined by NMR spectroscopy^29^ and also rationalize experimental data on chameleonicity^27^ or cell permeability.^29^ While MD simulations and the subsequent analysis of data are computationally resource-demanding, the current and recently reported^27,29^ results indicate that MD simulations could be valuable for property optimization of PROTACs in the later stages of lead optimization.

## Supplementary Material



## Data Availability

Original NMR spectra (FIDs) for PROTACs **1** and **2** and the structure files for the ensemble of PROTAC **1** in DMSO-*d*_6_/D_2_O are available free of charge on Zenodo as DOI: 10.5281/zenodo.14033616.

## Notes

Methods, purity analyses, and HRMS for **1** and **2**, computational analyses of MD simulations, 800 MHz ^1^H NMR data for **1** and **2**, and NAMFIS analysis for **1** (PDF)

## Abbreviations

bRo5: beyond rule of 5
CRBN: cereblon
ETR: EPSA to TPSA ratio
ERK5: extracellular signal-regulated kinase 5
FaSSIF: fasted-state simulated intestinal fluid
FeSSIF: fed-state simulated intestinal fluid
IMHB: intramolecular hydrogen bond
MEC: minimum-energy conformation
MD: molecular dynamics
NAMFIS: NMR analysis of molecular flexibility in solution
NOE: nuclear Overhauser effect
PAMPA: parallel artificial membrane permeability assay
POI: protein of interest, PROTAC, proteolysis-targeting chimera
*R*_gyr_: radius of gyration
PSA: polar surface area
SA 3D NPSA: solvent-accessible 3D nonpolar surface area
SA 3D PSA: solvent-accessible 3D polar surface area
TPSA: topological polar surface area
VHL: von Hippel–Lindau
