# Supplementary material for: Linker-Determined Folding and Hydrophobic Interactions Explain a Major Difference in PROTAC Cell Permeability
Source: ACS Med Chem Lett. 2025 Mar 17;16(4):681–7. doi: 10.1021/acsmedchemlett.5c00068 (PMC11995226; doi:10.1021/acsmedchemlett.5c00068)
Supplement: Supplementary file 1 [file ml5c00068_si_001.pdf]

SUPPORTING INFORMATION

Linker determined folding and hydrophobic interactions explain a major difference in PROTAC cell permeability

Vasanthanathan Poongavanam,[a,*] Stefan Peintner,[a] Yordanos Abeje,[a] Florian Kölling,[b] Daniel Meibom,[b] Mate Erdelyi[a] and Jan Kihlberg[a,*]

[a] Department of Chemistry - BMC, Uppsala University, Box 576, 75123 Uppsala, Sweden
[b] Bayer AG, Drug Discovery Sciences, 42113 Wuppertal, Germany

Corresponding authors
Vasanthanathan Poongavanam, vasanthanathan.poongavanam@kemi.uu.se, ORCID: 0000-0002-8880-9247
Jan Kihlberg, jan.kihlberg@kemi.uu.se, ORCID: 0000-0002-4205-6040

Contents

1. Experimental and Computational Methods

2. Characterization of the Purity and Identity of PROTACs **1** and **2**

3. Computational Analyses of MD Simulations of PROTACs **1** and **2**

4. Conformational Analysis of PROTAC **1** in DMSO-d_6:D_2O Solution

5. NMR Studies of PROTAC **2** in DMSO-d_6:D_2O Solution

6. References

1. Experimental and Computational Methods

Fasted and Fed State Simulated Intestinal Fluid (FaSSIF and FeSSIF) Solubility. Suspensions of amorphous **1** and **2** were stirred at 37 °C in FaSSIF and FeSSIF for 24 h ± 4 h prior to filtration and analysis of the filtrate. The solubility was determined via HPLC-MS analysis of the filtrate using a calibration curve (external standard).

EPSA. EPSA retention times for **1** and **2** were determined at Sygnature Discovery. In brief, the EPSA assay was performed on a Waters Acquity UPC2 system equipped with a PDA and a QDa detector, using a Phenomenex Chirex (S)-VAL and (R)-NEA LC column (50 x 4.6 mm). A gradient of MeOH, containing 20 mM ammonium formate, in CO_2 was used as eluent. EPSA retention times were calculated based on those of a standard mixture.[1]

Molecular Dynamics Simulations. The structures of PROTACs **1** and **2** were built using the Maestro module from the Schrödinger suite.[2] Before initiating the MD simulations, the geometries of **1** and **2** were optimized with Gaussian (version 16),[3] employing the HF method with the 6-31G** basis sets. Further refinement of the geometries was performed using the M06-2X functional and the 6-31+G** basis sets. Atomic charges for **1** and **2** were determined through electrostatic potential (ESP) fitting using the RESP procedure, as implemented in the Merz−Singh−Kollman scheme,[4] via the Antechamber tool.[5] MD simulations were then conducted in triplicate for **1** and **2** using the Amber software (version 18).[6]

The force field parameters used in the MD simulations of **1** and **2** were derived from the general Amber force field (GAFF), with GAFF atom types assigned using the Antechamber tool. Solvation was performed using TIP3P water molecules (to simulate the extra- and intracellular environments), adding approximately 1500 water molecules with a 10 Å buffer distance between the edges of the truncated octahedron box. For simulations in chloroform (to simulate the environment in the interior of the cell membrane), approximately 3000 chloroform molecules ($\varepsilon = 4.8$, frcmod.chcl3) were added, with a 30 Å buffer distance between the edges of the truncated octahedron box.

The topology parameters and coordinate input files were generated using the *tleap* tool from the Amber package.[6] Periodic boundary conditions were applied to mitigate edge effects during the MD simulations, which consisted of four stages: minimization, thermalization,

equilibration, and production runs. Energy minimization was performed in two steps. In the first step, the system (ligand and explicit water or chloroform molecules) was minimized using the steepest descent method with heavy atoms restrained for up to 1000 cycles. In the second step, the entire system underwent energy minimization without positional restraints for 200 cycles. Thermalization involved generating initial velocities at 100 K using a Maxwell-Boltzmann distribution and gradually increasing the temperature to 300 K under constant volume conditions over a 200 ps MD simulation. Once thermalized, the system was equilibrated at a constant temperature of 300 K and pressure of 1 bar using the Berendsen coupling algorithm,[7] followed by a 500 ps MD simulation. The production phase consisted of 100 ns MD simulations (run in triplicate), during which 30,000 snapshots were collected and analyzed. The SHAKE algorithm[8] was applied to constrain all bonds involving hydrogen atoms.

Trajectory analysis was performed using the *CPPTRAJ* module[9] from the Amber tool.[10] The total solvent accessible 3D surface area (SA 3D SA) and the solvent accessible 3D polar surface area (SA 3D PSA), defined using a solvent probe radius of 1.4 Å, and the R_{gyr} were calculated using VEGA ZZ (Release 3.2.3).[11] The solvent accessible 3D nonpolar surface area (SA 3D NPSA) was calculated by subtracting the SA 3D PSA from the total SA 3D SA surface area.

Boltzmann Population Analysis. To further assess the distribution of the conformations from the MD trajectories in $CHCl_3$ and water environments at room temperature, a Boltzmann population calculator was utilized to analyze the energy values obtained from the simulations. The default temperature was set at 298.15 K. Conformations with a population of 1% or less were excluded from further analysis.

NMR spectroscopy. The NMR spectra of PROTACs **1** and **2** were recorded in a solution of DMSO-d_6 and D_2O (9:1) at -10 and -5 °C for **1** and **2**, respectively, using an 800 MHz Bruker NEO NMR spectrometer equipped with a 5 mm TXO cryogenic probe. The compounds were assigned based on ^1H, TOCSY, COSY, HSQC, HMBC, and NOESY NMR spectra (Tables S2 and S8).

NOESY experiments were recorded with seven different mixing times (100, 200, 300, 400, 500, 600, and 700 ms). All spectra were recorded with 16 scans with 8192 and 512 points in the direct (F2) and indirect (F1) dimensions with a relaxation delay of 2.5 s. The spectra were

processed using the MestReNova version 14.3.0 software. The NOE peak intensities were normalized according to to $([cross\,peak_{ab} \times cross\,peak_{ba}]/[diagonal\,peak_a \times diagonal\,peak_b])^{0.5}$.[12] The initial rate approximation[13] was used to calculate interproton distances. A minimum of four mixing times with $R^2 \geq 0.91$ for the build-up rate (σ_{ab}) were used for distance calculation. Interproton distances were calculated based on the NOE build-up rates using the equation $r_{ab} = r_{ref}(\frac{\sigma_{ref}}{\sigma_{ab}})^{1/6}$, where r_{ab} is the distance between protons a and b, r_{ref} is the distance between two geminal methylene protons that are used as a reference (distance of 1.78 Å), and σ_{ref} and σ_{ab} are the slope of the NOE build-up curve for the reference and a, b protons (Tables S3 and S9).

Theoretical conformational ensemble and NAMFIS analysis of 1. The generation of the conformational ensemble used as input for the NAMFIS analysis of **1**, and the subsequent NAMFIS analysis, was performed as reported recently.[14]

Purity statement. PROTACs **1** and **2** are >95% pure by HPLC.

Safety statement. No unexpected or unusually high safety hazards were encountered.

2. Characterization of the Purity and Identity of PROTACs 1 and 2

Analytical Methods

LC/MS-Method 1: Instrument: SHIMADZU LCMS-2020 SingleQuad; Column: Chromolith@Flash RP-18E 25-2 MM; eluent A: water + 0.0375 vol % trifluoroaceticacid, eluent B: acetonitrile + 0.01875 vol % trifluoroacetic acid; gradient: 0-0.8 min, 5-95% B, 0.8-1.2 min 95% B; flow 1.5 ml/min; temperature: 50 °C; PDA: 220nm&254nm.
Single Mass Analysis (HR-MS) Instrument: Waters Time of Flight System (ToF), Electrospray Ionization (ESI).

LC/MS-Method 2: System MS: Waters TOF instrument; System UPLC: Waters Acquity I-CLASS; Column: Waters, HSST3, 2.1 x 50 mm, C18 1.8 µm; Eluent A: 1 l Water + 0.01% Formic acid; Eluent B: 1 l Acetonitrile + 0.01% Formic acid; Gradient: 0.0 min 2% B → 0.5 min 2% B → 7.5 min 95% B → 10.0 min 95% B; Oven: 50°C; Flow: 1.00 ml/min; UV-Detection: 210 nm

Single Mass Analysis (HR-MS) Instrument: Waters Time of Flight System (ToF), Electrospray Ionization (ESI).

Chromatograms and Spectra

3-methyl-N-{9-[(4-{1-[4-(trifluoromethoxy)benzoyl]piperidin-4-yl}pyrido[3,2-d]pyrimidin-7-yl)oxy]nonanoyl}-L-valyl-(4R)-4-hydroxy-N-[4-(4-methyl-1,3-thiazol-5-yl)benzyl]-L-prolinamide (**1**):

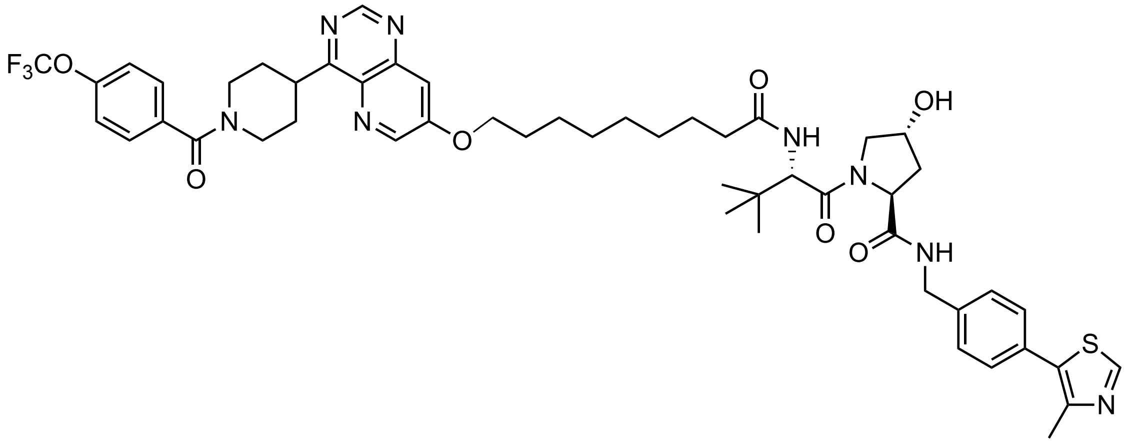

LC-MS (Method 1): R_t = 1.048 min; MS (ESIpos): m/z = 987 $[M-H]^+$

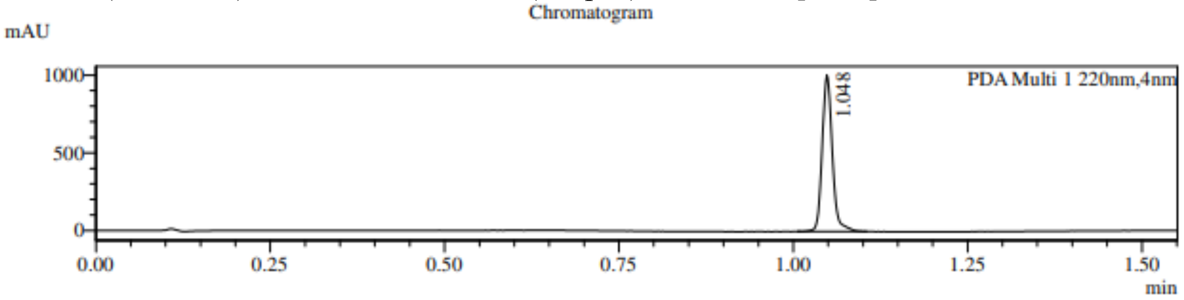

HRMS (ESI) m/z: calcd for $C_{51}H_{62}N_8O_7F_3S$ $[M+H]^+$, 987.4414; found, 987.4432

Single Mass Analysis
Tolerance = 2.0 PPM / DBE: min = -1.0, max = 50.0
Element prediction: Off
Number of isotope peaks used for i-FIT = 4

Monoisotopic Mass, Even Electron Ions
603 formula(e) evaluated with 1 results within limits (up to 50 best isotopic matches for each mass)
Elements Used:
C: 48-55 H: 20-100 N: 4-12 O: 4-12 F: 3-3 S: 0-1

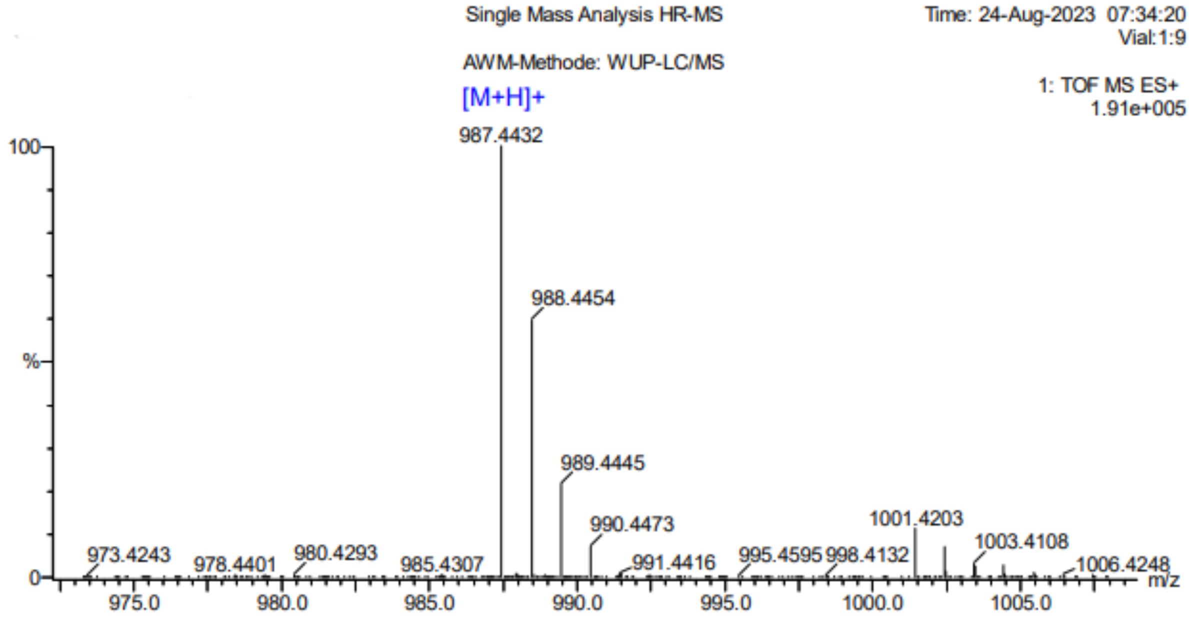

Minimum:				-1.0				
Maximum:		5.0	2.0	50.0				
Mass	Calc. Mass	mDa	PPM	DBE	i-FIT	Norm	Conf(%)	Formula
987.4432	987.4414	1.8	1.8	23.5	481.0	n/a	n/a	C51 H62 N8 O7 F3 S

Summenformelvorschlag

3-methyl-N-[3-(2-{2-[(4-{1-[4-(trifluoromethoxy) benzoyl]piperidin-4-yl}pyrido[3,2-d]pyrimidin-7-yl)oxy]ethoxy}ethoxy)propanoyl]-L-valyl-(4R)-4-hydroxy-N-[4-(4-methyl-1,3-thiazol-5-yl)benzyl]-L-prolinamide (**2**):

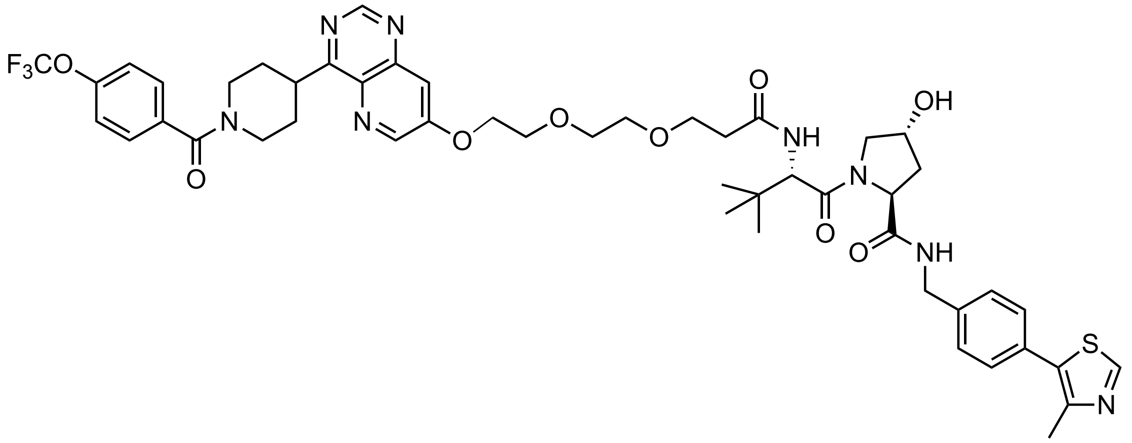

LC-MS (Method 2): R_t = 4.38 min; MS (ESIpos): m/z = 991 $[M+H]^+$

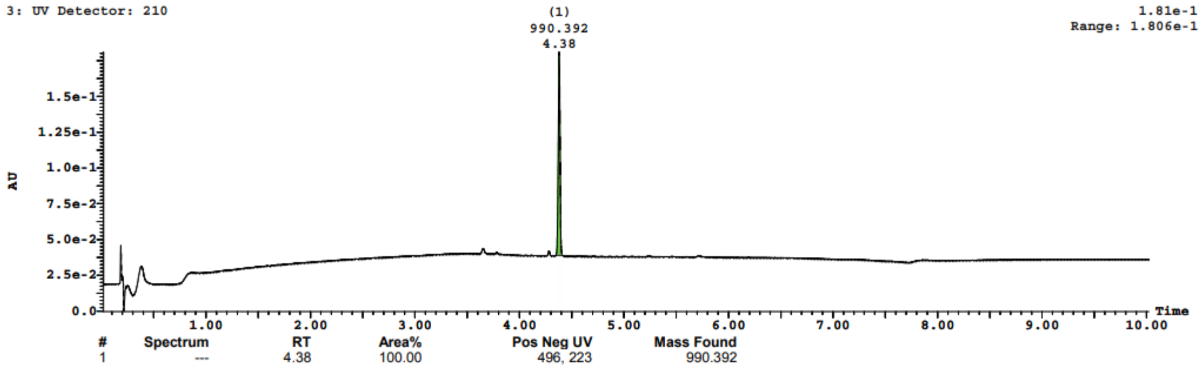

HRMS (ESI) m/z: calcd for $C_{49}H_{58}N_8O_9F_3S$ $[M+H]^+$, 991.4006; found, 991.4006

Single Mass Analysis
Tolerance = 2.0 PPM / DBE: min = -1.0, max = 30.0
Element prediction: Off
Number of isotope peaks used for i-FIT = 3

Monoisotopic Mass, Even Electron Ions
350 formula(e) evaluated with 1 results within limits (up to 50 closest results for each mass)
Elements Used:
C: 45-55 H: 10-100 N: 0-10 O: 0-10 F: 3-3 S: 1-1

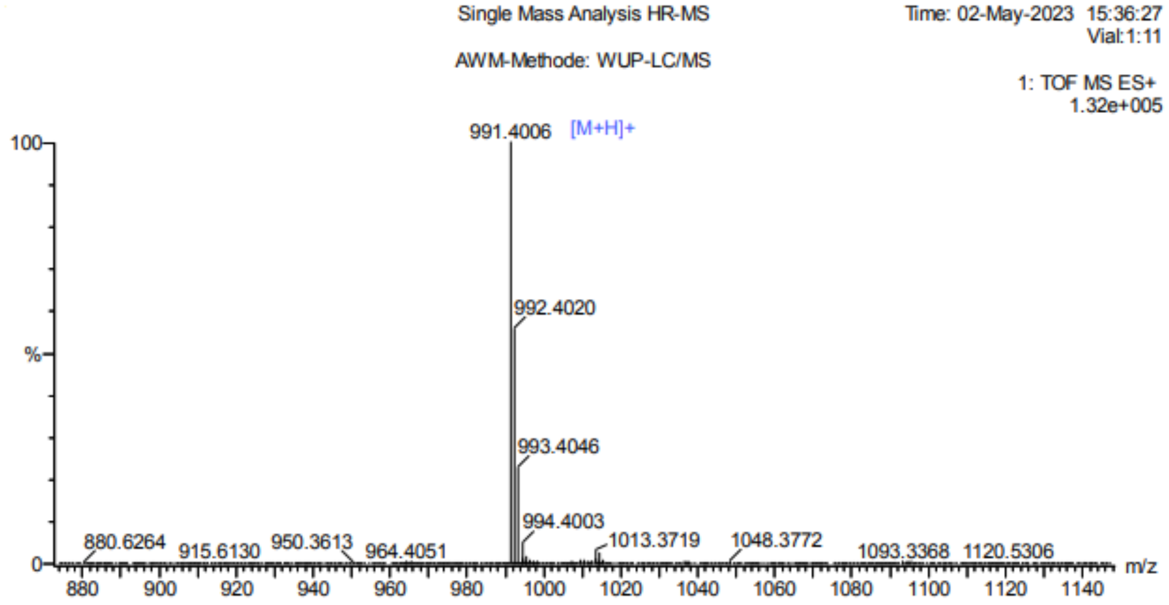

Minimum:				-1.0				
Maximum:		5.0	2.0	30.0				
Mass	Calc. Mass	mDa	PPM	DBE	i-FIT	Norm	Conf(%)	Formula
991.4006	991.4000	0.6	0.6	23.5	257.1	n/a	n/a	C49 H58 N8 O9 F3 S

Summenformelvorschlag

3. Computational Analyses of MD Simulations of PROTACs 1 and 2

Figure S1. RMSD time series for the MD simulations of PROTACs **1** and **2** relative to the initial position. Averaged RMSD (blue lines) over the three replicates, gray shading represents standard deviations.

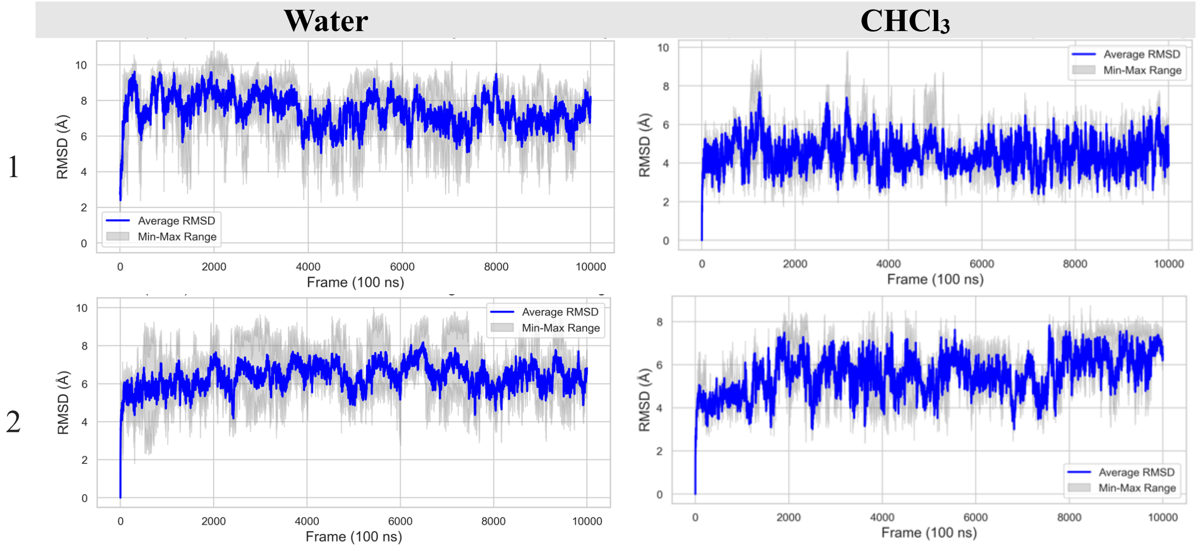

Figure S2. Density properties [SA 3D PSA (Å^2) versus R_{gyr} (Å)] for PROTACs **1** and **2**. Data has been plotted for the three independent replicates of the 100 ns simulations. Densely sampled property space is indicated by the landscape contours.

PROTAC 1 in Water

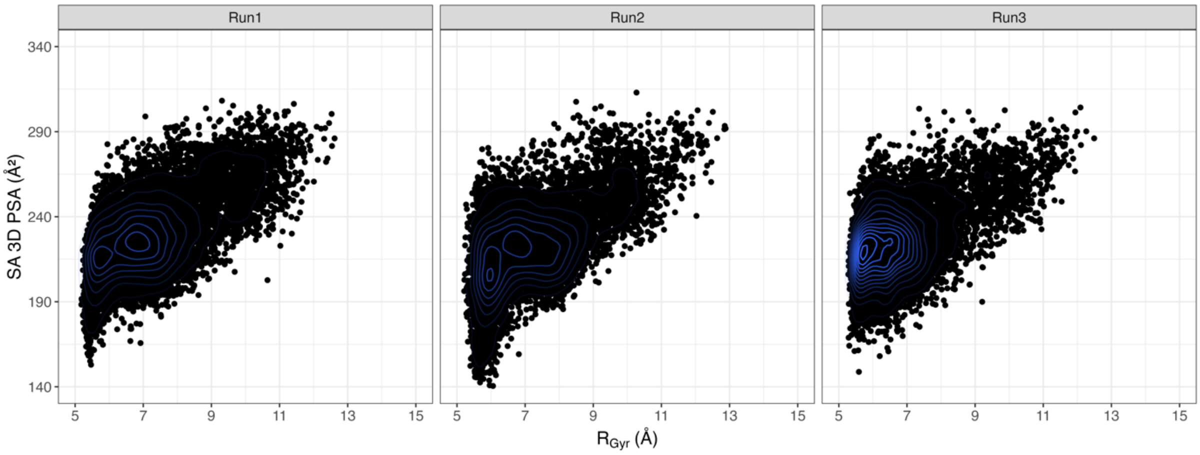

PROTAC 2 in Water

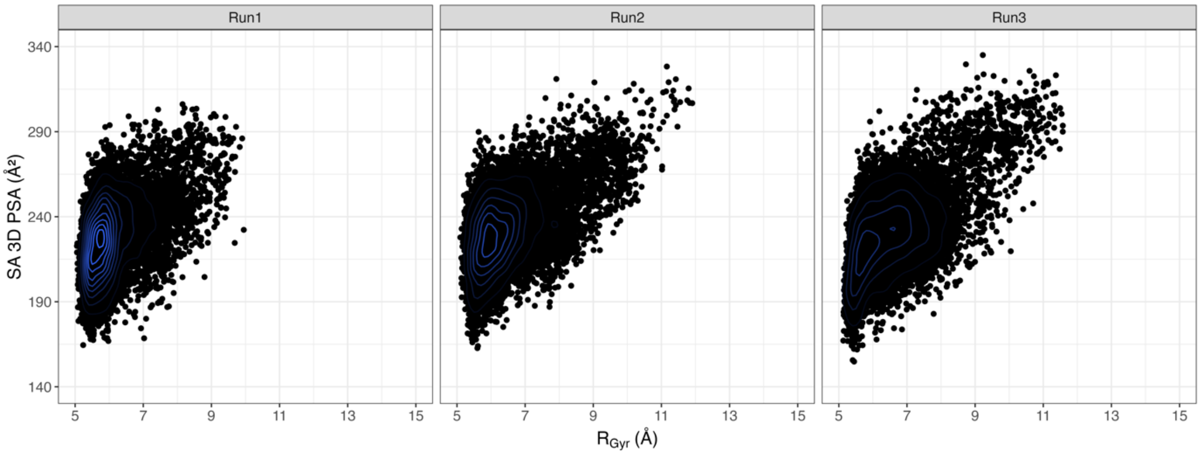

PROTAC 1 in $CHCl_3$

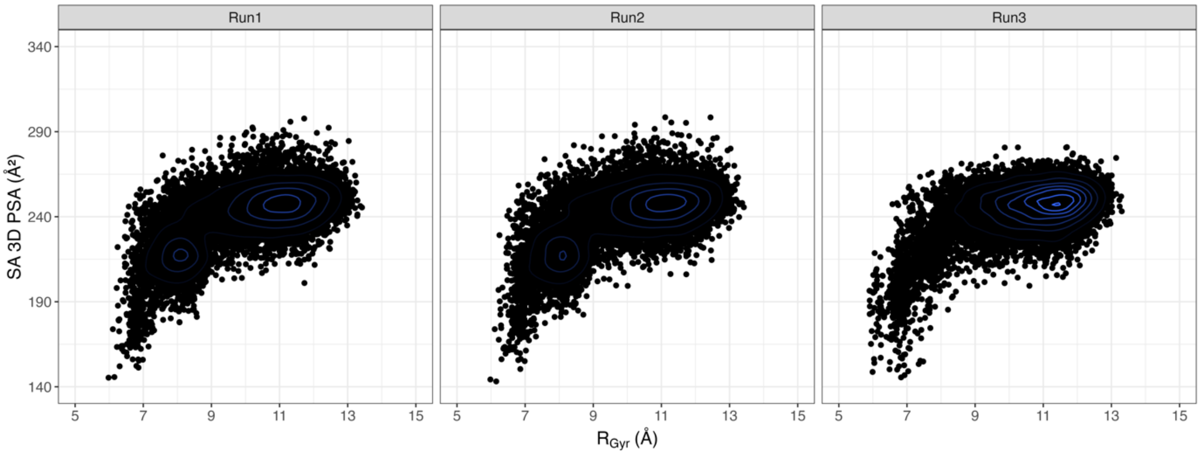

PROTAC 2 in $CHCl_3$

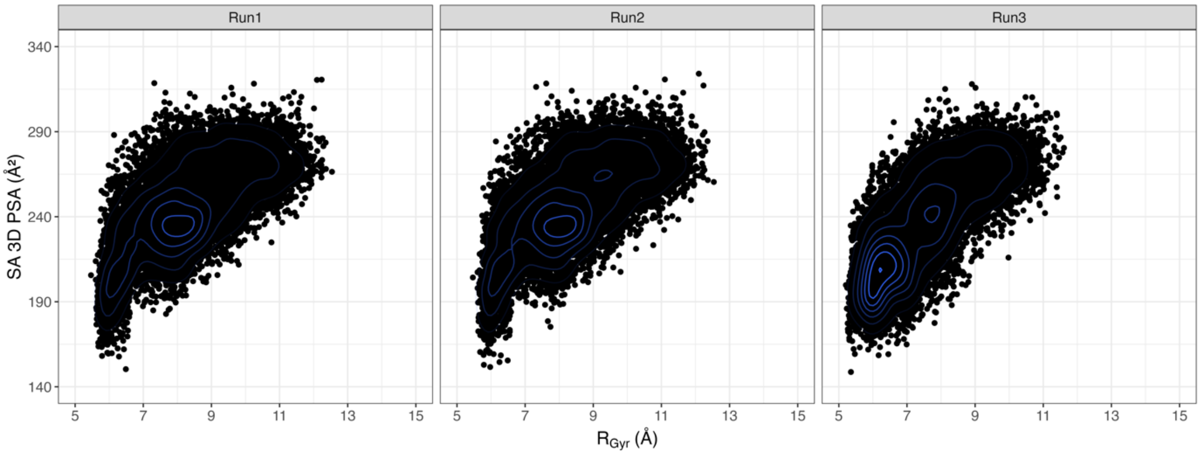

Figure S3. Solvent accessible 3D polar surface area (SA 3D PSA) time series for PROTACs **1** and **2**. Averaged (blue lines) over the three independent replicates, gray shading represents standard deviations.

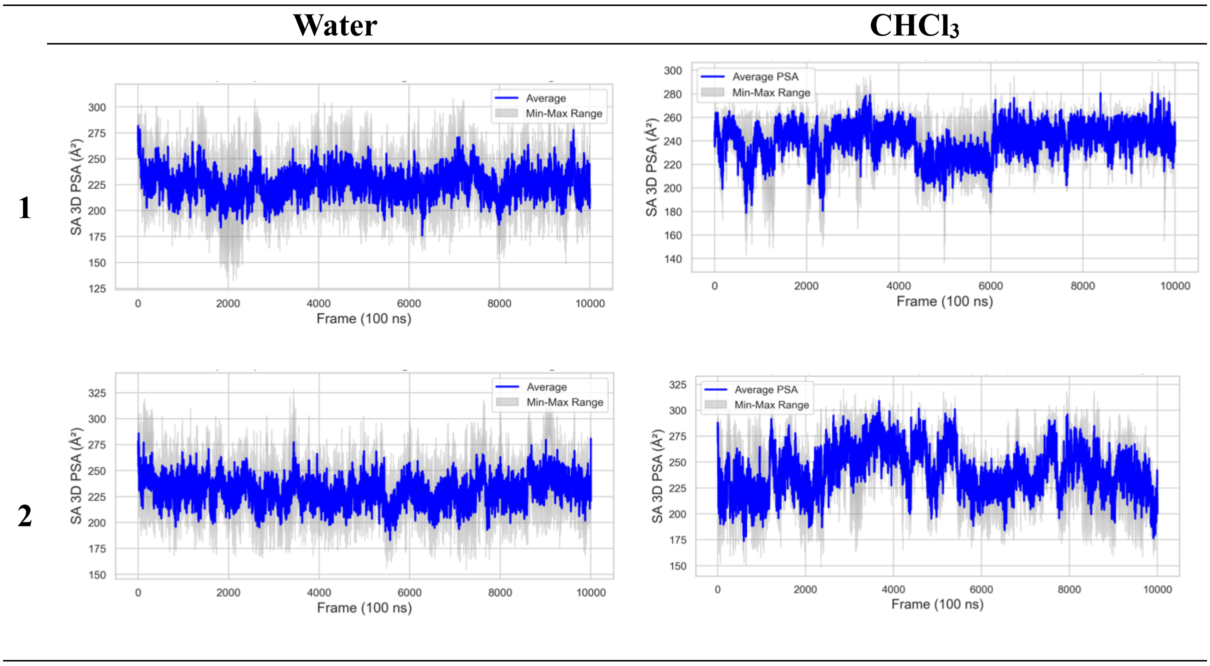

Figure S4. Radius of gyration (R_{gyr}) time series for PROTACs **1** and **2**. Averaged (blue lines) over the three independent replicates, gray shading represents standard deviations.

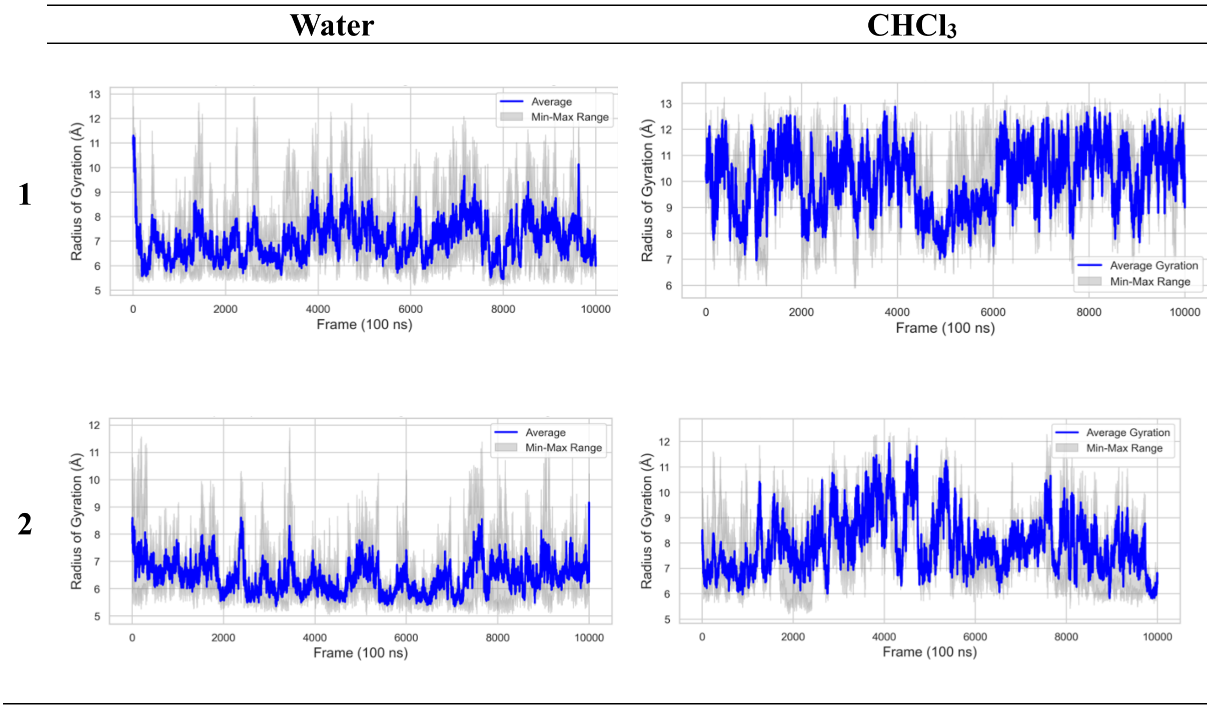

Figure S5. Conformational ensemble of PROTAC **1** in water after clustering and Boltzmann population analysis.

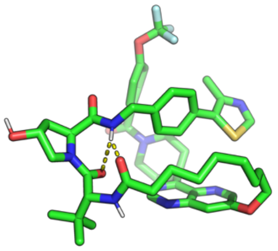	
**Conformation 16100 (100%)**	

Figure S6. Conformational ensemble of PROTAC **1** in $CHCl_3$ after clustering and Boltzmann population analysis.

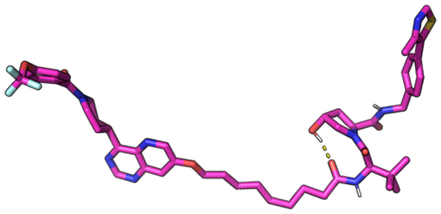	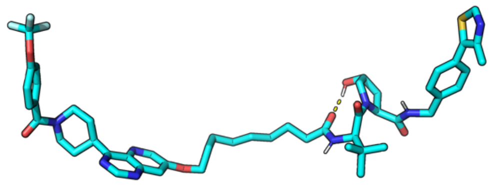
**Conformation 28997 (94%)**	**Conformation 29353 (6 %)**

Figure S7. Conformational ensemble of PROTAC **2** in water after clustering and Boltzmann population analysis

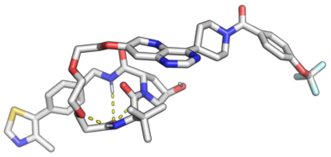	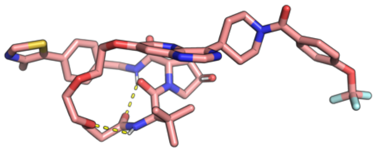
**Conformation 25741 (79%)**	**Conformation 3348 (20%)**

Figure S8. Conformational ensemble of PROTAC **1** in $CHCl_3$ after clustering and Boltzmann population analysis.

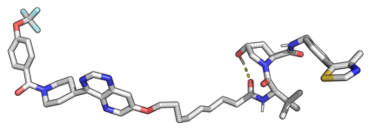	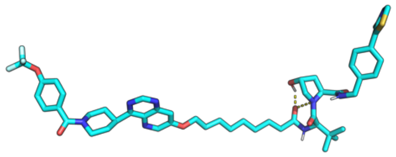	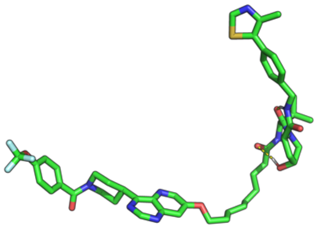
**Conformation 19261 (91%)**	**Conformation 17113 (2%)**	**Conformation 16911 (2%)**

Table S1. Conformation-dependent molecular descriptors for the low energy conformations from the MD simulations for PROTAC **1** and **2**

Compound	Solvent	Conf. ID	SA 3D PSA ($Å^2$)	SA 3D NPSA	R_{gyr} (Å)	Popl.(%) [Bw]
1	$CHCl_3$	16911	229.2	1242.8	9.6	2
1	$CHCl_3$	17113	262.7	1215.2	12.0	2
1	$CHCl_3$	19261	250.3	1202.6	10.7	91
1	Water	16100	182.4	857.2	5.3	100
2	$CHCl_3$	24776	185.2	945.5	6.7	20
2	$CHCl_3$	25741	205.9	936.8	7.0	79
2	Water	25540	191.1	835.0	5.4	82
2	Water	3349	191.1	817.9	5.3	15

[BW] *Boltzmann weighted*

4. Conformational Analysis of PROTAC 1 in DMSO-d_6:D_2O Solution

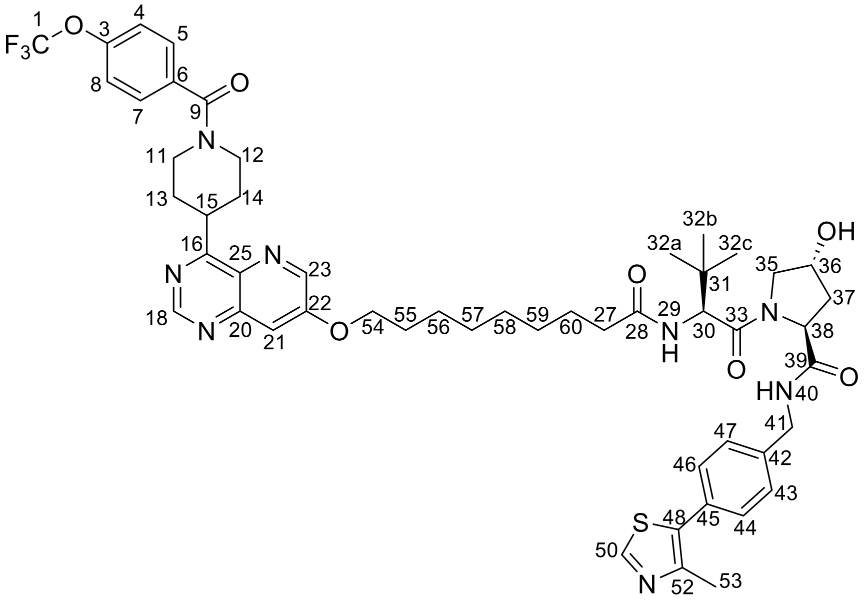

Figure S9. Structure and atom-numbering of PROTAC **1**

Table S2. ^{1}H- and ^{13}C-NMR chemical shift assignments of PROTAC **1** in DMSO-d_6:D_2O (9:1) at -10 °C.

Assigned	^{13}C (ppm)	^{1}H (ppm)	Assigned	^{13}C (ppm)	^{1}H (ppm)
	**POI**		32 a, b, c	26.9	0.85
1	120.6 (q)	-	33	170.3	-
3	149.4	-	35', 35''	57.1	3.62
4-8	121.9	7.45	36	69.4	4.31
5-7	129.7	7.55	37'	38.3	1.85
6	135.9	-	37''		2.02
9	168.7	-	38	59.4	4.37
11'	42.3	2.97	39	173.0	-
11''		4.60	40-NH	-	8.77
12'	47.9	3.29	41'	42.2	4.16
12''		3.65	41''		4.41
13'	30.8	1.80	42	140.0	-
13''		1.93	43, 47	128.0	7.40
14', 14''	31.2	1.79	44, 46	129.3	7.34
15	37.4	4.33	45	130.1	-
16	173.3	-	48	132.0	-
18	156.1	9.15	50	152.4	8.95
20	148.1	-	52	148.3	-
21	126.1	7.68	53	16.5	2.39
22	158.6	-		**LINKER**	
23	146.9	8.78	54', 54''	69.6	4.17
25	132.9	-	55', 55''	28.8	1.76

	**VHL**		56’, 56’’	26.0	1.39
27’	35.3	2.07	57’, 57’’	29.3	1.27
27’’		2.25	58’, 58"	29.2	1.22
28	173.4	-	59’, 59"	29.1	1.19
29-NH	-	8.07	60’	26.0	1.41
30	57.0	4.49	60’’		1.49
31	35.9	-			

Table S3. Interproton distances, r_{AB} (Å) derived from NOE build-up curves in DMSO-d_6:D_2O (9:1) at -10 °C for PROTAC **1** that were included in the NAMFIS analysis.

**No.**	**Proton A**	**Proton(s) B**		**δ_A (ppm)**	**δ_B (ppm)**	**σ**	**R^2**	**Distance, r_{AB} (Å)**
1	**38**	**57'**	**57"**	4.37	1.27	2.983x10^{-6}	0.94	5.01
2	**30**	**57'**	**57"**	4.49	1.27	1.489x10^{-5}	0.96	3.83
3	**21**	**57'**	**57"**	7.68	1.27	8.276x10^{-5}	0.96	4.66
4	**30**	**55'**	**55"**	4.49	1.76	6.013x10^{-6}	0.97	4.46
5	**29-NH**	**43**	**47**	8.07	7.40	9.591x10^{-6}	0.98	4.13
6	**21**	**55'**	**55"**	7.68	1.76	2.568x10^{-4}	0.98	3.20
7	**29-NH**	**35'**	**35"**	8.07	3.62	1.441x10^{-4}	0.98	2.63
8	**38**	**29**		4.37	8.07	1.873x10^{-5}	0.98	3.69
9	**60"**	**38**		1.49	4.37	2.001x10^{-6}	0.95	5.36
10	**60"**	**30**		1.49	4.49	2.845x10^{-5}	0.97	3.44
11	**27"**	**38**		2.25	4.37	5.070x10^{-6}	0.99	4.59
12	**27"**	**30**		2.25	4.49	8.740x10^{-5}	0.99	2.86
13	**27'**	**30**		2.08	4.49	8.064x10^{-5}	0.98	2.89
14	**60"**	**57'**	**57"**	1.49	1.27	2.834x10^{-4}	0.99	3.09
15	**11'**	**44**	**46**	2.97	7.34	1.648x10^{-6}	0.98	5.53
16	**11'**	**43**	**47**	2.97	7.40	1.345x10^{-6}	0.96	5.73
17	**37"**	**43**	**47**	2.02	7.40	3.475x10^{-5}	0.98	3.33
18	**30**	**43**	**47**	4.49	7.40	1.119x10^{-5}	0.96	4.02
19	**29-NH**	**55'**	**55"**	8.07	1.76	1.059x10^{-5}	0.93	4.06
20	**30**	**38**		4.49	4.37	8.825x10^{-5}	0.99	2.85
Ref	**37’**	**37”**		1.85	2.02	0.001489	0.96	1.78

Table S4. Results of the Monte Carlo Molecular Mechanics (MCMM) conformational searches for PROTAC **1**.

Solvent	Force Field	Number of conformations	
		Total[a]	Final ensemble[b]
**$CHCl_3$**	OPLS	358	337
	OPLS-2005	1071	
	OPLS4	1610	
	AMBER*	1631	
	MMFF	2436	
**H_2O**	OPLS	523	
	OPLS-2005	946	
	OPLS4	972	
	AMBER*	608	
	MMFF	865	

[a] Total number of unique conformations found. The global minimum was found for all investigated compounds at least 5 times. [b] Total number of unique conformations obtained after redundant conformer elimination with the root-mean-square deviation cutoff 3.0 Å for heavy atoms.

Table S5. Result of the NMR Analysis of Molecular Flexibility in Solution (NAMFIS) analysis for PROTAC **1** in DMSO-d$_6$:D_2O (9:1) at -10 °C.

Conf. No.	%	Conf. No.	%
1	10	6	11
2	7	7	9
3	6	8	5
4	29	9	14
5	6	10	3

Table S6. Experimentally determined and back-calculated (NAMFIS) interproton distances (Å) of PROTAC **1** in DMSO-*d*$_6$:D_2O (9:1) at -10 °C.

PROTAC 1	
Exp.	Calc.
5.01	4.85
3.83	3.85
4.66	4.70
4.46	4.45
4.13	4.19
3.20	3.20
2.63	2.64
3.69	3.57
5.36	5.41
3.44	3.46
4.59	4.57
2.86	2.73

2.89	2.75
3.09	3.18
5.53	5.50
5.73	5.76
3.33	3.45
4.02	4.13
4.06	4.18
2.85	2.80

Figure S10. Structures and populations of the conformations in the ensemble determined by NAMFIS analysis for PROTAC **1** in DMSO-d_6:D_2O (9:1) at -10 °C.

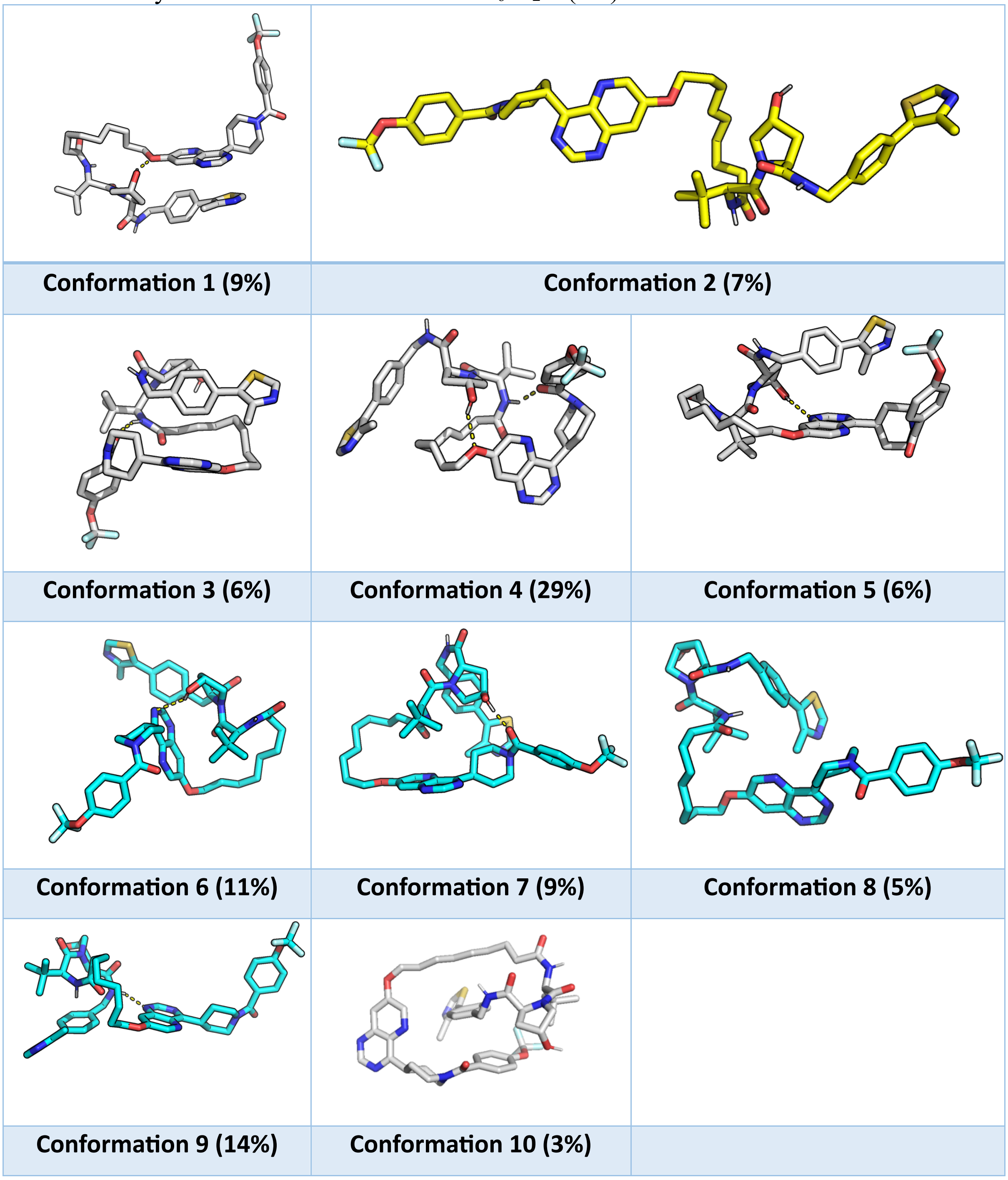

Table S7. Conformation-dependent molecular descriptors for the conformations in the ensemble determined by NAMFIS analysis for PROTAC **1** in DMSO-d_6:D_2O (9:1) at -10 °C.

Conf. No.	SA 3D SA[a] (Å²)	SA 3D NPSA[a] (Å²)	SA 3D PSA[a] (Å²)	SA 3D PSA[b] (Å²)	R_{gyr} [a] (Å)
**1**	1134.24	941.49	192.75	163.15	7.01
**2**	1330.23	1054.74	275.50	238.13	9.18
**3**	1045.08	863.99	181.09	148.18	5.48
**4**	1094.30	897.25	197.05	159.90	5.82
**5**	1149.76	926.75	223.01	180.74	6.11
**6**	1162.02	941.40	220.61	180.84	6.31
**7**	1098.08	886.33	211.74	170.85	5.94
**8**	1181.39	942.32	239.07	199.27	6.75
**9**	1187.56	983.24	204.33	165.93	7.12
**10**	1022.58	773.59	248.98	229.29	5.38

[a] Calculated using VEGA (Release 3.2.3).[11]
[b] Calculated using PyMol as reported previously.[15]
Abbreviations: SA 3D SA: total solvent accessible 3D surface area; SA 3D NPSA: solvent accessible 3D nonpolar surface area; SA 3D PSA; solvent accessible 3D polar surface area; R_{gyr}: radius of gyration.

Figure S11. Correlation between the solvent accessible 3D polar surface area (SA 3D PSA) calculated with PyMol[15] and VEGA.[11]

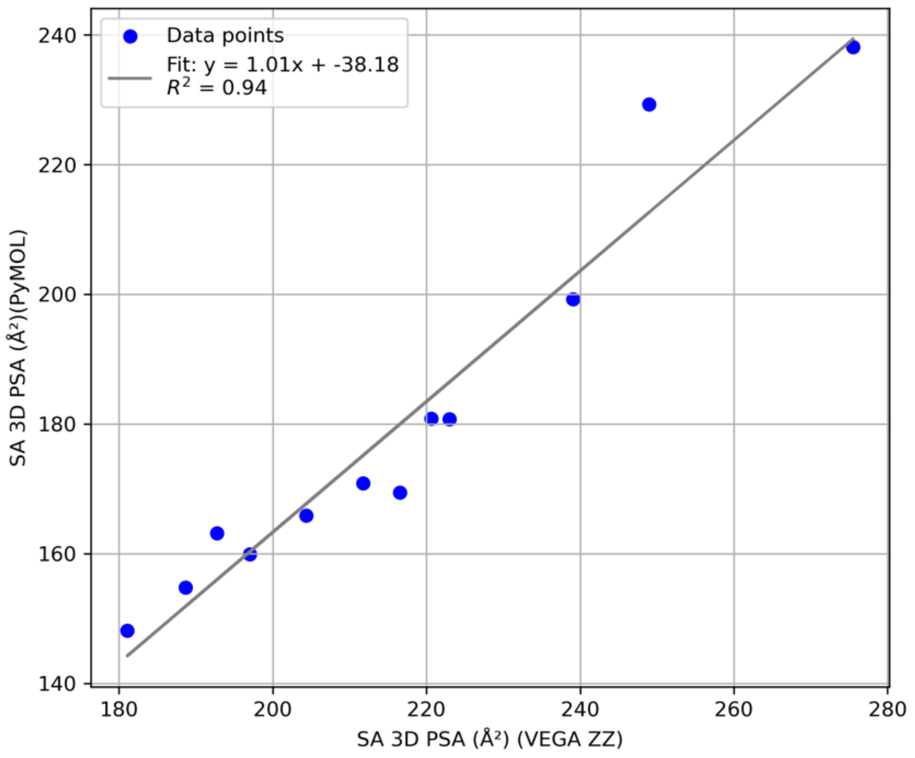

5. NMR Studies of PROTAC 2 in DMSO-d_6:D_2O Solution

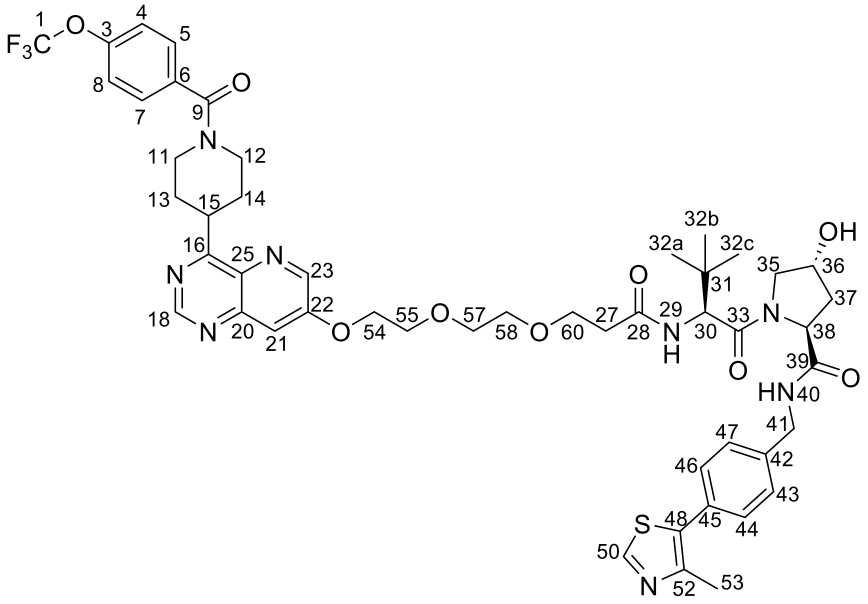

Figure S12. Structure and atom-numbering of PROTAC **2**

Table S8. ^{1}H-NMR chemical shift assignments of PROTAC **2** in DMSO-d$_6$:D_2O (9:1) at -5 °C.

Proton(s) no.	δ(^{1}H)
4, 8	7.39
5, 7	7.32
15	4.33
18	9.16
21	7.73
23	8.80
27′	2.51
27′′	2.31
29	8.08
30	4.50
32	3.90
35	3.61
36	4.31
37′	2.03
37′′	1.85
38	4.38
40	8.76
41′	4.42
41′′	4.16
43, 47	7.45
44, 46	7.55
50	8.93
53	0.85

54	4.32
55	3.80
57	3.56
58	3.49
60	3.56

Table S9. Interproton distances, r_{AB} (Å) derived from NOE build-up curves in DMSO-d_6:D_2O (9:1) at -5 °C for PROTAC **2**

#	Proton 1	Proton 2	δA (ppm)	δB (ppm)	σ	R2	Distance (Å)
1	**18**	**21**	9.16	7.73	1E-05	0.91	3.99
2	**23**	**57, 60**	8.8	3.56	8.4E-06	0.99	4.14
3	**23**	**53**	8.8	0.85	7.7E-06	0.99	4.20
4	**23**	**21**	8.8	7.73	1.9E-05	0.98	3.61
5	**23**	**55**	8.8	3.8	2.4E-05	0.99	3.48
6	**23**	**36, 54**	8.8	4.31	5E-05	0.99	3.08
7	**40**	**37″**	8.76	1.85	0.00018	0.95	2.48
8	**40**	**53**	8.76	0.85	1.2E-05	0.99	3.90
9	**40**	**35**	8.76	3.61	3.7E-05	0.96	3.23
10	**40**	**38**	8.76	4.38	0.0008	0.93	1.94
11	**40**	**37′**	8.76	2.03	0.00019	0.97	2.47
12	**40**	**41″**	8.76	4.16	0.00043	0.95	2.15
13	**29**	**27″**	8.08	2.31	0.00044	0.99	2.14
14	**29**	**57, 60**	8.08	3.56	0.0001	0.98	2.74
15	**29**	**53**	8.08	0-85	0.0002	0.99	2.44
16	**29**	**27′**	8.08	2.51	5.9E-05	0.96	2.99
17	**29**	**30**	8.08	4.50	0.00017	0.99	2.51
18	**21**	**53**	7.73	0.85	9.3E-06	0.99	4.07
19	**21**	**36, 54**	7.73	4.31	0.00062	0.99	2.02
20	**21**	**57, 60**	7.73	3.56	2.4E-05	0.96	3.47
21	**21**	**55**	7.73	3.80	0.00021	0.98	2.42
22	**44, 46**	**43, 47**	7.55	7.45	0.00037	0.99	2.21
23	**4, 8**	**35**	7.39	3.61	7.9E-06	0.97	4.18
24	**4, 8**	**41″**	7.39	4.16	0.00014	0.99	2.59
25	**4, 8**	**37″**	7.39	1.85	2.7E-05	0.99	3.41
26	**4, 8**	**37′**	7.39	2.03	2.4E-05	0.99	3.47
27	**4, 8**	**53**	7.39	0.85	2.7E-05	0.99	3.40
28	**4, 8**	**5, 7**	7.39	7.32	0.00042	0.99	2.16
29	**5, 7**	**37″**	7.32	1.85	8.6E-06	0.99	4.13
30	**5, 7**	**37′**	7.32	2.03	6.8E-06	0.98	4.29
31	**5, 7**	**41″**	7.32	4.16	3.4E-05	0.99	3.28
32	**5, 7**	**53**	732	0.85	1.8E-05	0.99	3.64
33	**30**	**35**	4.5	3.61	0.00079	0.99	1.94

34	**30**	**37´´**	4.5	1.85	0.00014	0.98	2.59
35	**30**	**36, 54**	4.5	4.31	0.00012	0.99	2.65
36	**30**	**53**	4.5	0.85	0.00034	0.99	2.24
37	**38**	**37´´**	4.38	1.85	0.00029	0.99	2.30
38	**38**	**37´**	4.38	2.03	0.00037	0.99	2.21
39	**38**	**35**	4.38	3.61	8.1E-05	0.97	2.84
40	**38**	**36, 54**	4.38	4.31	5.2E-05	0.97	3.05
41	**38**	**41´´**	4.38	4.16	0.00023	0.97	2.39
42	**36, 54**	**35**	4.31	3.61	0.00027	0.99	2.33
43	**36, 54**	**37´**	4.31	2.03	0.00033	0.99	2.25
44	**36, 54**	**38**	4.31	438	5.2E-05	0.97	3.05
45	**36, 54**	**37´´**	4.31	1.85	0.00035	0.99	2.22
46	**36, 54**	**53**	4.31	0.85	6.2E-05	0.99	2.97
47	**36, 54**	**57, 60**	4.31	3.56	4.9E-05	0.97	3.09
48	**36, 54**	**55**	4.31	3.80	0.00035	0.99	2.23
49	**41´´**	**38**	4.16	4.38	0.00023	0.97	2.39
50	**55**	**36, 54**	3.80	4.31	0.00035	0.99	2.23
51	**55**	**57, 60**	3.80	3.56	0.00018	0.99	2.48
52	**35**	**36, 54**	3.80	4.31	0.00027	0.99	2.33
53	**35**	**53**	3.80	0.85	0.00018	0.99	2.48
54	**35**	**37´**	3.80	2.03	0.00022	0.99	2.40
55	**35**	**37´´**	3.80	1.85	0.00029	0.99	2.30
56	**57, 60**	**36, 54**	3.56	4.31	4.9E-05	0.97	3.09
57	**57, 60**	**53**	3.56	0.85	5.2E-05	0.99	3.05
58	**57, 60**	**27´**	3.56	2.51	1.7E-05	0.97	3.67
59	**57, 60**	**27´´**	3.56	2.31	0.00026	0.99	2.34
60	**37´**	**36, 54**	2.03	4.31	0.00033	0.99	2.25
61	**37´´**	**36, 54**	1.85	4.31	0.00035	0.99	2.22
62	**53**	**36, 54**	0.85	4.31	5.1E-05	0.94	3.06
Ref	**41´**	**41´´**	4.42	4.16	0.00133	0.99	1.78

6. References

(1) Goetz, G. H., Philippe, L., Shapiro, M. J. EPSA: A novel supercritical fluid chromatography technique enabling the design of permeable cyclic peptides. *ACS Medicinal Chemistry Letters* **2014**, 5, 1167-1172

(2) *Maestro, Schrödinger, LLC, New York, NY.*; 2022.

(3) *Gaussian 16 Rev. C.01*; Wallingford, CT, 2016.

(4) Singh, U. C., Kollman, P. A. An approach to computing electrostatic charges for molecules. *Journal of Computational Chemistry* **1984**, 5, 129-145

(5) Wang, J. M. W., W.; Kollman, P. A. . Antechamber: An accessory software package for molecular mechanical calculations. In *Abstr. Pap. Am. Chem. Soc.*, 2001; Vol. 222.

(6) D.A. Case, H. M. A., K. Belfon, I.Y. Ben-Shalom, J.T. Berryman, S.R. Brozell, D.S. Cerutti, T.E. Cheatham, III, G.A. Cisneros, V.W.D. Cruzeiro, T.A. Darden, N. Forouzesh, M. Ghazimirsaeed, G. Giambaşu, T. Giese, M.K. Gilson, H. Gohlke, A.W. Goetz, J. Harris, Z. Huang, S. Izadi, S.A. Izmailov, K. Kasavajhala, M.C. Kaymak, A. Kovalenko, T. Kurtzman, T.S. Lee, P. Li, Z. Li, C. Lin, J. Liu, T. Luchko, R. Luo, M. Machado, M. Manathunga, K.M. Merz, Y. Miao, O. Mikhailovskii, G. Monard, H. Nguyen, K.A. O'Hearn, A. Onufriev, F. Pan, S. Pantano, A. Rahnamoun, D.R. Roe, A. Roitberg, C. Sagui, S. Schott-Verdugo, A. Shajan, J. Shen, C.L. Simmerling, N.R. Skrynnikov, J. Smith, J. Swails, R.C. Walker, J. Wang, J. Wang, X. Wu, Y. Wu, Y. Xiong, Y. Xue, D.M. York, C. Zhao, Q. Zhu, and P.A. Kollman. Amber. 2024.

(7) Berendsen, H. J. C., Postma, J. P. M., van Gunsteren, W. F., DiNola, A., Haak, J. R. Molecular dynamics with coupling to an external bath. *The Journal of Chemical Physics* **1984**, 81, 3684-3690

(8) Ryckaert, J.-P., Ciccotti, G., Berendsen, H. J. C. Numerical integration of the cartesian equations of motion of a system with constraints: molecular dynamics of n-alkanes. *Journal of Computational Physics* **1977**, 23, 327-341

(9) Roe, D. R., Cheatham, T. E., 3rd. PTRAJ and CPPTRAJ: Software for Processing and Analysis of Molecular Dynamics Trajectory Data. *Journal of Chemical Theory and Computation* **2013**, 9, 3084-3095

(10) Case, D. A., Aktulga, H. M., Belfon, K., Cerutti, D. S., Cisneros, G. A., Cruzeiro, V. W. D., Forouzesh, N., Giese, T. J., Gotz, A. W., Gohlke, H., Izadi, S., Kasavajhala, K., Kaymak, M. C., King, E., Kurtzman, T., Lee, T. S., Li, P., Liu, J., Luchko, T., Luo, R., Manathunga, M., Machado, M. R., Nguyen, H. M., O'Hearn, K. A., Onufriev, A. V., Pan, F., Pantano, S., Qi, R.,

Rahnamoun, A., Risheh, A., Schott-Verdugo, S., Shajan, A., Swails, J., Wang, J., Wei, H., Wu, X., Wu, Y., Zhang, S., Zhao, S., Zhu, Q., Cheatham, T. E., 3rd, Roe, D. R., Roitberg, A., Simmerling, C., York, D. M., Nagan, M. C., Merz, K. M., Jr. AmberTools. *Journal of Chemical Information and Modeling* **2023**, 63, 6183-6191

(11) Pedretti, A., Villa, L., Vistoli, G. VEGA: a versatile program to convert, handle and visualize molecular structure on Windows-based PCs. *Journal of Molecular Graphics and Modelling* **2002**, 21, 47-49

(12) Hu, H., Krishnamurthy, K. Revisiting the initial rate approximation in kinetic NOE measurements. *Journal of Magnetic Resonance* **2006**, 182, 173-177

(13) Vögeli, B. The nuclear Overhauser effect from a quantitative perspective. *Progress in Nuclear Magnetic Resonance Spectroscopy* **2014**, 78, 1-46

(14) Abeje, Y. A., Wieske, L. H. E., Poongavanam, V., Maassen, S., Atilaw, Y., Cromm, P., Lehmann, L., Erdelyi, M., Meibom, D., Kihlberg, J. Impact of linker composition on VHL PROTAC cell permeability. *Journal of Medicinal Chemistry* **2025**, 68, 638−657

(15) Danelius, E., Poongavanam, V., Peintner, S., Wieske, L. H. E., Erdélyi, M., Kihlberg, J. Solution conformations explain the chameleonic behavior of macrocyclic drugs. *Chemistry European Journal* **2020**, 26, 5231-5244
